# Impact of Tenascin-C on Radiotherapy in a Novel Syngeneic Oral Squamous Cell Carcinoma Model With Spontaneous Dissemination to the Lymph Nodes

**DOI:** 10.3389/fimmu.2021.636108

**Published:** 2021-07-05

**Authors:** Caroline Spenlé, Thomas Loustau, Hélène Burckel, Gilles Riegel, Chérine Abou Faycal, Chengbei Li, Alev Yilmaz, Luciana Petti, Fanny Steinbach, Constance Ahowesso, Camille Jost, Nicodème Paul, Raphael Carapito, Georges Noël, Fabienne Anjuère, Nathalie Salomé, Gertraud Orend

**Affiliations:** ^1^ INSERM U1109-MN3T, The Microenvironmental Niche in Tumorigenesis and Targeted Therapy, Strasbourg, France; ^2^ Université de Strasbourg, Strasbourg, France; ^3^ Fédération de Médecine Translationnelle de Strasbourg (FMTS), Strasbourg, France; ^4^ INSERM U1109, The Tumor Microenvironment Group, Strasbourg, France; ^5^ Institut de Cancérologie de Strasbourg Europe (ICANS), UNICANCER, Paul Strauss Comprehensive Cancer Center, Radiobiology Laboratory, Université de Strasbourg, Strasbourg, France; ^6^ Université Côte d’Azur, CNRS, IPMC, Valbonne-Sophia Antipolis, France; ^7^ Platform GENOMAX, INSERM UMR_S 1109, Faculté de Médecine, Fédération Hospitalo-Universitaire OMICARE, LabEx TRANSPLANTEX, Strasbourg, France; ^8^ Institut de Cancérologie Strasbourg Europe (ICANS), UNICANCER, Department of Radiation Oncology, Strasbourg, France

**Keywords:** radiotherapy, oral squamous carcinoma, tumor microenvironment, tenascin-C, syngeneic animal model, immune suppression

## Abstract

Radiotherapy, the most frequent treatment of oral squamous cell carcinomas (OSCC) besides surgery is employed to kill tumor cells but, radiotherapy may also promote tumor relapse where the immune-suppressive tumor microenvironment (TME) could be instrumental. We established a novel syngeneic grafting model from a carcinogen-induced tongue tumor, OSCC13, to address the impact of radiotherapy on OSCC. This model revealed similarities with human OSCC, recapitulating carcinogen-induced mutations found in smoking associated human tongue tumors, abundant tumor infiltrating leukocytes (TIL) and, spontaneous tumor cell dissemination to the local lymph nodes. Cultured OSCC13 cells and OSCC13-derived tongue tumors were sensitive to irradiation. At the chosen dose of 2 Gy mimicking treatment of human OSCC patients not all tumor cells were killed allowing to investigate effects on the TME. By investigating expression of the extracellular matrix molecule tenascin-C (TNC), an indicator of an immune suppressive TME, we observed high local TNC expression and TIL infiltration in the irradiated tumors. In a TNC knockout host the TME appeared less immune suppressive with a tendency towards more tumor regression than in WT conditions. Altogether, our novel syngeneic tongue OSCC grafting model, sharing important features with the human OSCC disease could be relevant for future anti-cancer targeting of OSCC by radiotherapy and other therapeutic approaches.

## Introduction

Head and neck squamous cell carcinoma (HNSCC) is the 7th most frequent cancer with a low percentage of 5-year survival (1). HNSCC in the oral cavity, lip, tongue and upper throat, coined oral squamous cell carcinoma (OSCC), can metastasize to regional lymph nodes and the lung (2). Exposure to tobacco, bethel nut and alcohol represent high risk factors for developing OSCC (1, 3).

Apart from surgical removal of cancer tissue in OSCC, radiotherapy is the most common treatment (4, 5). Radiotherapy is used in 70% of cancer patients and includes high energy rays (photons, protons or charged particles) where the total dose varies between 50 and 70 Gy, with a daily fractionation of 1.8–2 Gy (5). Absorption of these rays induces DNA double strand breaks and reactive oxygen species causing a tumoricidal effect that is largely dependent on an anti-tumor immune response (6, 7). Dying tumor cells can generate neoantigens that activate the immune system involving dendritic cells (DC), macrophages and other immune subtypes (8). Radiotherapy can act as a two-edged sword by activating and inhibiting the immune defense against tumor cells, respectively (9, 10).

The extracellular matrix (ECM) molecule tenascin-C (TNC) is highly expressed in malignant tumors including HNSCC and plays multiple roles in the tumor microenvironment (TME) promoting cancer progression and metastasis (11–13). TNC promotes tumor cell survival, proliferation, invasion, formation of leaky blood vessels and lung metastasis as demonstrated in spontaneous tumor models with high TNC in comparison to engineered low TNC (14–16). Moreover, TNC forms local niches within the tumor where stromal and immune cells are enriched, regulating cell behavior (14, 15, 17). Recently, by using mice expressing or lacking TNC, we have shown that TNC orchestrates an immune-suppressive TME in the carcinogen 4-nitroquinoline-1-oxide (4NQO)-induced OSCC model (18).

In the past several OSCC tumor grafting models have been established from 4NQO-induced tongue, lip and esophageal squamous cell carcinomas that were used in drug targeting (19–23). Some of these models showed high resemblance with human papilloma virus (HPV)-negative OSCC which was recently confirmed at mutation level (24). However, in none of these models the impact of radiotherapy on the TME has been addressed.

Here, we established a novel syngeneic OSCC model derived from a 4NQO-induced OSCC in the tongue that showed similarities with HPV-negative HNSCC by recapitulating mutations seen in human tumors, constitutive transforming growth factor beta (TGFβ) signaling, abundant TIL in TNC-rich stroma, and spontaneous tumor cell dissemination to the local lymph nodes. Most importantly, this model was sensitive to radiotherapy and revealed an impact of TNC on tumor regression. This model does allow not only to investigate tumor regression by radiotherapy but could be useful for assessing effects of radiotherapy on the TME. Finally, our model could be relevant for future anti-cancer targeting of OSCC by radiotherapy and/or other therapeutic approaches.

## Material and Methods

### Cell Culture

OSCC2 cells were established from a murine 4NQO induced tongue tumor arising in a female C57Bl/6J mouse (18) and cultured in DMEM-F12 with 4.5 g/L glucose, 10% FBS, 1% penicillin–streptomycin (Sigma, P4333), 40 µg/ml gentamicin (Dutscher, L0012-100) and 0.4 μg/ml hydrocortisone (Sigma, H4001). Cells were checked for the absence of mycoplasms (once every two months, PlasmoTest, Invivogen, rep-pt1). OSCC2 cells (5 × 10^6^) were subcutaneously engrafted into the back of a C57Bl/6J mouse for 4 weeks before extraction of the tumor, treatment with collagenase as described (18) and explanting cells into cell culture dishes. After three passages *in vitro*, 5 × 10^6^ cells were again grafted into the neck of a C57Bl/6J mouse which induced a tumor from which cells were explanted in cell culture as described, giving rise to the cell line OSCC13. For bioluminescence experiments, OSCC13 cells were infected with lentiviral particles carrying a luciferase reporter gene. In brief, lentiviral particles were established with ViraPower™ Lentiviral Expression Systems (Invitrogen) and the plasmid pLenti-CMV-LUC (Addgene, #21474) in HEK293T cells (ATCC). OSCC13 cells were then infected with the lentiviral particles and cells stably expressing the luciferase reporter were established by selection with 10 µg/ml of blasticidin (InvivoGen) giving rise to the OSCC13-LUC cell line.

### Orthotopic Grafting of OSCC13 Cells in the Tongue and Bioluminescence Detection

Nude mice (8 weeks of age) or WT and TNCKO (C57Bl/6J) mice (bred in house) were grafted in the first third part of the tongue. In particular, 3 × 10^6^ OSCC13 cells in 10 µl PBS were injected using a U-100 insulin syringe (BD Micro-Fine) in C57Bl/6J mice. Some 1 × 10^6^ cells OSCC13-LUC (luciferase expression vector expressing cells) were similarly grafted. Tumors were visible 2 weeks upon engraftment and mice were sacrificed for analysis at 3 weeks (bioluminescence experiment), 2 and 4 weeks (NIR) or 4 weeks (IR). For irradiation analysis, a 2 Gy unique dose of photons was delivered two weeks after tumor cell engraftment (3 × 10^6^ cells). Mice were sacrificed two weeks later and tissue was analyzed by staining. For bioluminescence detection, a RediJect D-Luciferin Ultra Bioluminescent Substrate (Perkinelmer 770505) solution at 30 mg/ml was injected intraperitoneally 7 min before imaging. Images were acquired for 5 min using a live imager (NightOwl, Berthold). All mice were housed and handled according to the guidelines of INSERM and the ethical committee of Alsace, France (CREMEAS) (Directive 2010/63/EU, 01386.02, C-67-482-033 on the protection of animals used for scientific purposes).

### Immune Cell Preparation From Tumors and Cytokine Production

OSCC13 tumor tissues were cut in small pieces and then treated with a solution of collagenase IV (1 mg/ml; Sigma-Aldrich, France) and DNase (0.2 mg/ml; Roche Diagnostics, France) at 37°C for 20 min under constant shaking. Cell suspensions from the OSCC13 tongue tumors (400,000 cells/well) were stimulated with anti-CD3/CD28 dynabeads (Gibco #11452D) at a bead-to-cell ratio of 1:1 for 24 h according to the provider’s instructions (Becton Dickinson). Supernatants were collected and assessed for the indicated cytokines using the CBA technology (BD Biosciences, France).

### Immunostaining

For immunofluorescence staining (IF), unfixed frozen 8 μm tumor sections or cells fixed with 2% paraformaldehyde (PFA) followed by permeabilization in 0.1% TritonX-100/PBS for 10 min were directly incubated overnight with the primary antibodies (**Supplementary Table S1**). Secondary antibodies conjugated with Alexa 488, Cy3 or Cy5 were used (**Supplementary Table S1**). Dapi (Sigma D9542) was used to visualize nuclei. After embedding in FluorSave Reagent (Calbiochem, 345789), sections were examined using a Zeiss Axio Imager Z2 microscope. Pictures were taken with an AxioCam MRm (Zeiss) camera and were analyzed using the Axiovision software. Control sections were processed as mentioned above with omission of the primary antibodies. The image acquisition setting (microscope, magnification, light intensity, exposure time) was kept constant per experiment and in between experimental conditions (comparison NIR with IR tumors) or was adjusted to get the best image (all other stainings).

### Real-Time Quantitative PCR

Cells were dissolved in the TRIzol reagent (Invitrogen, 12044977) for total RNA extraction. RNA quality was confirmed by optical density measurement. cDNA was synthesized from 1 μg of total RNA using random primers and Moloney murine leukemia virus reverse transcriptase (MultiScribe, Applied Biosystems, 10117254). The cDNA was used for qRTPCR in an Mx3005P Real-Time PCR System (ThermoFisher Scientific). Reactions were carried out in duplicate for all conditions using a Sybr Green Master mix (ThermoFisher Scientific, 4344463) or Fast Taqman mix (ThermoFisher Scientific, 4444557) and expression of mouse Gapdh mRNA (Life Technology, 433764T) was used as endogenous control in the comparative cycle threshold method (2^−^ΔΔ^Ct^). Primer sequences used are listed in **Supplementary Table S2**.

### RNAseq Analysis

RNA from OSCC13 cells (grown for 24 h in DMEM/10% FCS, N = 2) was isolated using the RNeasy Mini Kit (Qiagen). For each sample, quality control was carried out and assessed with the NGS Core Tools FastQC (http://www.bioinformatics.babraham.ac.uk/projects/fastqc/). Sequence reads were mapped using STAR (25). The total mapped reads were finally available in a BAM (Binary Alignment Map) format for variant calling. Variant calling was performed using the ultra-sensitive variant caller VarDict (26) where only variants with a PASS status were selected. At last, the variants were annotated by the Ensembl Variant Effect Predictor tool (27). Mutation signatures were obtained using the computational framework SigProfiler (https://www.mathworks.com/matlabcentral/fileexchange/38724-sigprofiler). Gene mutation analyses were performed by comparing data from OSCC13 cells with that from publicly available HNSCC dataset from the TCGA consortium (28) and 4MOQ cell lines previously described (24). In addition, p53 mutation patterns observed in the characterized HNSCC samples from TCGA and OCSS13 cell lines were represented and compared using the “lolipop” mutation pattern generator (29). From the list of genes expressed by OSCC13 cells, an over-representation analysis (ORA) was performed in order to determine the gene sets statistically over-represented (**Supplementary Table S3**). Visualization of genes and GO terms was performed using Webgestalt program (30) and Gene Ontology database (31). Multiple testing Benjamini–Hochberg correction was performed and an False-discovery rate threshold was applied (FDR ≤0.05). The original data are available at ArrayExpress accession *E-MTAB-10614*.

### Irradiation of Tumor Cells and Tumor Mice

Cells seeded in 6-well plates (Falcon, 353046) were exposed, at room temperature, to gamma irradiation, with single doses of 2, 4, 6, 8 or 10 Gy. A ^137^Cs γ-irradiator (Biobeam GM 8000, GSM GmbH, Leipzig, Germany) was used in the Paul Strauss Center/Institut de Cancérologie Strasbourg Europe (Strasbourg, France) at a dose rate of 2.5 Gy/min. Cells in control flasks were sham irradiated. Tumor bearing (WT and TNCKO) mice, 2 weeks after engrafting of 3 × 10^6^ OSCC13 cells in the tongue, were irradiated under anesthesia (ketamine 100 mg/kg and xylazine 10 mg/kg) with the irradiator Biobeam GM 8000 with a single fraction of 2 Gy at dose rate of 2.5 Gy/min. The whole body of the mouse, apart from the front of the head, was protected with a lead shield to avoid radiation toxicity. Eyes were protected with a moisturizing cream. No toxic effect was seen in non-tumor bearing mice as assessed by quantification of white and red blood cells numbers, respectively.

### Cell Proliferation Assay

*In vitro* determination of OSCC2 and OSCC13 cell growth was performed using a resazurin assay (Interchim (UP669413 Upti-blue, Montluçon, France). Cells were seeded in 96-well plates at a density of 1 × 10^3^ cells per well in 100 μl and incubated for 24 h. Subsequently, cells were irradiated with single irradiation doses and incubated at 37°C for 1, 2, 3 or 6 days. Subsequently, 20 μl (10% of final volume) of resazurin were added to each well and cells were incubated at 37°C for four additional hours. The fluorescence of each well was measured at 560–590 nm using a Synergy™ microplate reader (Biotek, Winooski, USA). Results were expressed in arbitrary units of fluorescence (AUF) after subtraction of the blank value (medium only). All experiments were repeated at least three times independently.

### Clonogenic Survival Assay

Cells were seeded in 6-well plates (Falcon, 353046) at a density of 2 × 10^5^ cells per well and allowed to adhere overnight in standard culture conditions (DMEM with 10% FBS). Cells were then exposed to irradiation and collected 24 h after irradiation. Cells were trypsinized and enumerated using a Countess^®^ cell counter (Countess, Invitrogen, Carlsbad, USA). Then, cells were seeded in fresh medium and plated at two different dilutions into 6-well plates. The seeding densities used were 100 and 200 cells for the control non-irradiated condition, 200 and 400 cells after 2 Gy, 400 and 800 after 4 Gy, 750 and 1500 after 6 Gy, 1,500 and 3,000 after 8 Gy and 3,000 and 6,000 cells after 10 Gy. Thirteen days later, clones were stained using 0.05% crystal violet (Sigma-Aldrich, C0775) in a 5% ethanol solution and positive colonies containing more than 50 cells were scored. The plating efficiency (PE) and then the surviving fractions (SF) were calculated. The plating efficiency (PE) is defined as the ratio of the number of colonies formed for each condition to the number of cells seeded for each condition. Furthermore, the surviving fraction (SF) is expressed as the ratio of the plating efficiency after treatment to plating efficiency without treatment (non-irradiated control). Survival curves were plotted using surviving fractions for the different doses. Treatments were performed in triplicate and the experiments were repeated four times independently.

### Statistical Analysis

For all data, Gaussian distribution was tested. When data followed a Gaussian distribution, statistical differences were analyzed by unpaired t-test (with Welch’s correction in case of unequal variance) or ANOVA one-way with Tukey post-test. Otherwise, the Mann–Whitney test or a non-parametric ANOVA followed by Dunns post-test were used to verify significance of the observed differences. For clonogenic survival assay, data were compared pairwise with a Student–Newman–Keuls test. All statistical analyses were performed using the GraphPad Prism 5.02 software. Mean ± SEM. p values <0.05 were considered as statistically significant, *p <0.05; **p <0.01; ***p <0.001.

## Results

### Establishment of the OSCC13 Cell Line From a 4NQO-Induced Tongue Tumor

**Figure 1:**
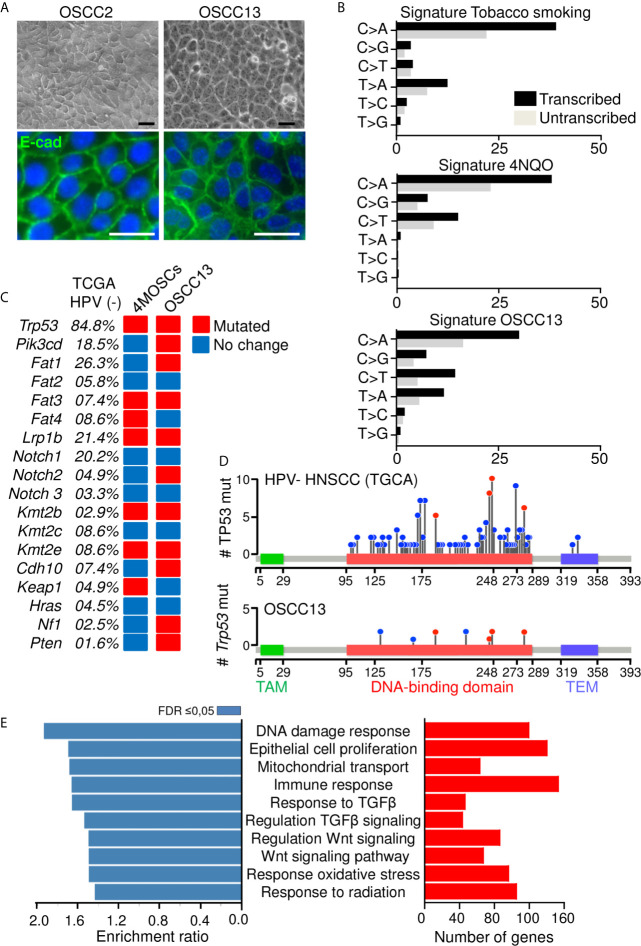
Phenotype of a new OSCC model. **(A)** Phase contrast and IF imaging of E-cadherin in OSCC2 and OSCC13 cells. Scale bar, 25 µm. **(B)** Percentage of somatic substitutions located in the translated or untranslated genome regions in patients with smoking-associated HNSCC (top (32), in 4NQO-derived lesions (middle (24); and in OSCC13 cells (bottom). **(C)** Graphical matrix representation of individual mutations in OSCC13 compared to 4MOSCs and smoking-associated HNSCC (24, 32). The most frequently observed alterations in the indicated genes of human HPV negative HNSCC (TGCA database) and the corresponding percentage of mutations in 4MOSCs and OSCC13 are shown. The presence (red) or absence (blue) of mutations are indicated for OSCC13 and the 4MOSC1–4 cells when at least two of the four cells showed the same phenotype. **(D, E)** RNA data analysis of cultured OSCC13 cells 24 h after plating. **(D)** Lollipop mutational plot of TP53 mutations in human HNSCC (HPV-negative) tumor samples from TCGA database (top) and mouse HNSCC cell line (OSCC13) (bottom). The frequency of mutations is represented by the height of the lollipop. The blue circles indicate mutations specific to human or mouse, and the red circles illustrate mutations common to human and mouse HNSCCs. TAM, Transactivation motif; TEM, Tetramerisation motif. **(E)** Over-represented gene ontology terms as determined by the Webgestalt program and Geneontology database. Bar charts show the enrichment ratio (blue) and expressed gene numbers (red) of the 10 most over-represented GO processes associated with FDR ≤ 0.05.

We established a murine OSCC tumor by exposing a C57Bl/6J mouse to the carcinogen 4-nitroquinoline-1-oxide (4NQO) in the drinking water for 20 weeks as recently described (18). Then cells were explanted giving rise to the epithelioid cell line OSCC2 as seen by phase contrast imaging and immunofluorescence (IF) staining for E-cadherin (**Figure 1A**). As OSCC2 cells generated tumors in the tongues of 60% of mice we sought to increase the grafting efficiency to 100% which was accomplished by a two times sub-cutaneous grafting of OSCC2 cells in the upper neck of C57Bl/6J mice. The arising OSCC13 cells were phenotypically indistinguishable from the OSCC2 cells as seen by phase contrast imaging and E-cadherin IF staining (**Figure 1A**). To compare the genetic differences and similarities between OSCC13 cells and human HNSCC tumors, we performed RNAseq analysis. By using SigProfiler program we compared the mutation rate in OSCC13 cells with that in tobacco smoking associated human HNSCC (32) and in 4NQO-induced tongue tumors (24). As described previously, cells extracted from 4NQO-induced tumors show remarkable similarity to human cancer cells (24). The mutational patterns induced by 4NQO and tobacco have similar higher mutation rate in translated than in untranslated regions and substitution type rates on each nucleotide. Only the T to A transition, frequently mutated in the tobacco signature is barely found in the 4NQO signature. Interestingly the OSCC13 signature presented nucleotide substitution rates recapitulating tobacco and 4NQO signatures with both high T to A and C to T substitution frequencies (**Figure 1B**). Investigation of mutations in 18 genes that are frequently mutated in human HPV-negative HNSCC and in the 4NQO-induced 4MOSC cells, revealed also high mutation rates in the OSCC13 cells (except for Fat4 and Keap1 that did not show mutations). Moreover, six other genes frequently affected in human OSCC, but not found to be altered in the 4MOSC cells, were also mutated in the OSCC13 cells (Pik3cd, Fat1, Notch2, Cdh10, Nf1 and Pten) suggesting that OSCC13 cells phenocopy characteristics described for human HNSCC (**Figure 1C**). TP53 encoding the tumor suppressor molecule p53 is frequently mutated in human tumors (84.8% of HPV-negative HNSCC (TGCA cohort)). We investigated hotspots of mutations in TP53 and observed seven mutations in the DNA binding domain with four in common to HPV-negative HNSCC (**Figure 1D**). Next, by over-representation analysis (ORA) we investigated gene expression of the cultured OSCC13 cells. We observed several immune modulating molecules such as *Cd274, Ctla4* and *Irf7* to be expressed by the cultured OSCC13 cells, mimicking HNSCC, 4NQO-induced tumors and the 4MOSC1-4 cells [**Supplementary Table S3** (18, 24, 33)]. Moreover, many molecules involved in the TGFβ (46 genes) and Wnt signaling pathways (68 genes) were significantly expressed suggesting intrinsic activation of these pathways again recapitulating features of human HNSCC [**Figure 1E** and **Supplementary Table S3** (34, 35)]. Altogether, we propose that the OSCC13 cells could be a relevant novel cellular model that recapitulates properties of human tobacco smoking associated HNSCC.

### OSCC13 Cells Induce Tumors Upon Grafting in the Tongue of Syngeneic Mice

**Figure 2:**
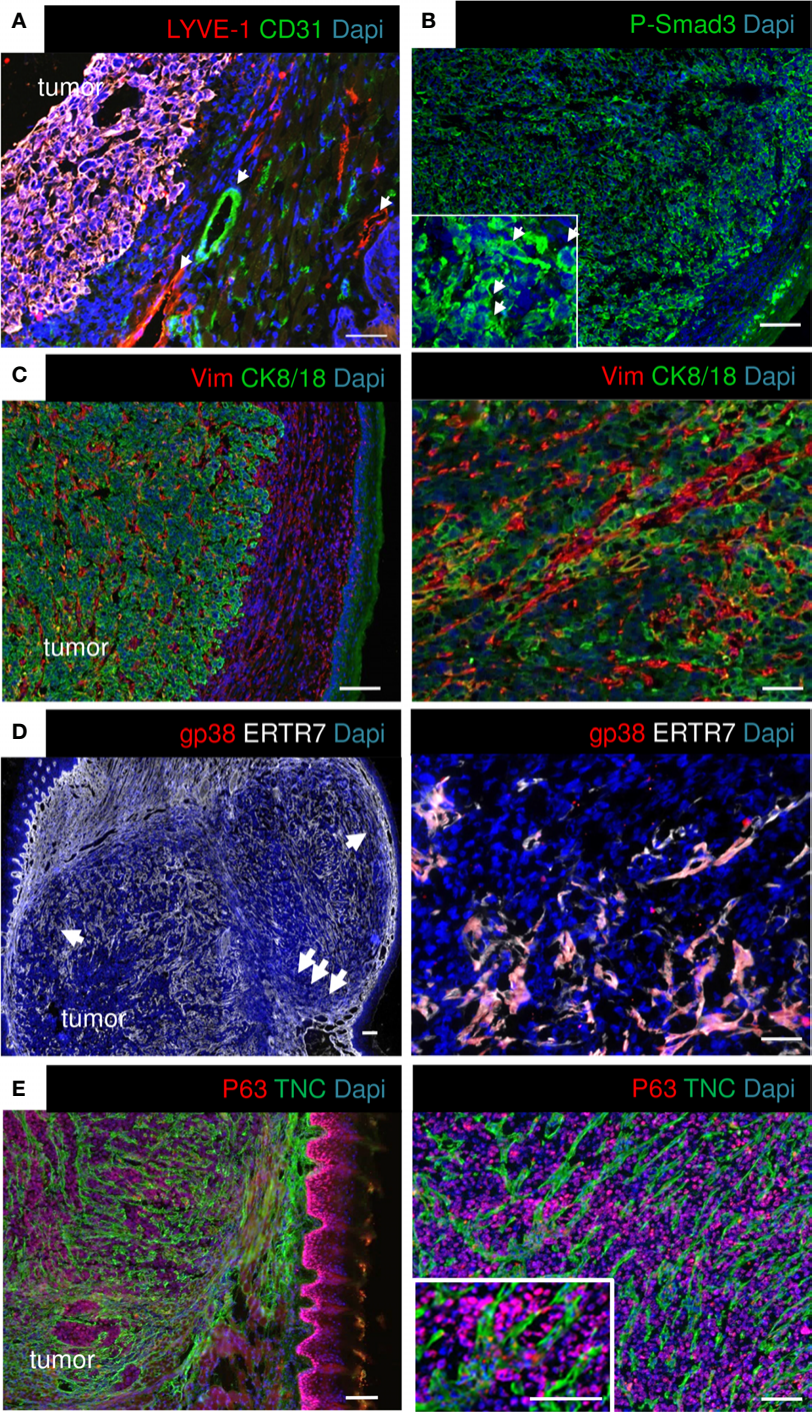
Characterization of OSCC13 tumors by immunostaining. 4 weeks syngeneic tongue tumors derived from 1 × 10^6^ engrafted OSCC13 cells were stained for the indicated molecules. Representative images are shown (N = 3). Scale bar, 100 µm. Arrows point at LYVE+ lymphatic and CD31+ blood vessels in the surroundings of the tumor mass **(A),** at P-Smad3+ cells (higher magnification inlet in **B**), and at ERTR7+ cells invading the tumor mass from the surrounding tissue **(D)**. **(C–E)**, scale bar, 500 μm (left). Right panels, higher magnification, scale bar, 100 μm **(C, E)**, 50 μm **(D)**.

Grafting OSCC13 cells underneath the mucosa induced tongue tumors that were collected and characterized by tissue staining with hematoxylin and eosin (H&E), and IF with antibodies against specific markers. We observed that tumors highly expressed TNC (**Supplementary Figures S1A, B**). Human OSCC tumors and 4NQO-induced tumors are highly vascularized (18, 36), therefore we stained for lymphatic (LYVE1) and blood vessels (CD31). We observed a high staining of LYVE1 and CD31 around but not inside the tumors which indicates that the angiogenic switch may not have occurred yet (**Figure 2A**). As the RNA seq analysis revealed constitutive TGFβ signaling in the tumor cells we investigated phosphorylation of Smad3 as indicator of pathway activation. Indeed, P-Smad3 was highly abundant however mostly cytoplasmic (**Figure 2B**). As TGFβ signaling can be activated in stromal, immune and cancer cells (34), future studies have to determine in which stromal cells this pathway is preactivated in the OSCC13 tumors. OSCC tongue tumors are mainly of epithelial origin (two over three patients) (37). We investigated the cellular properties by combined staining for CK8/18 and vimentin and noticed that the majority of cells inside the tumor mass expressed CK8/18 (**Figure 2C**). In addition, streaks of vimentin+ cells were seen to separate CK8/18+ tumor cell nests (**Figure 2C**). Vimentin+ cells likely represent carcinoma associated fibroblasts. Future studies have to reveal whether vimentin+ cells also derive from tumor cells having undergone epithelial-to-mesenchymal transition (EMT). As ERTR7+ fibroblast reticular cells (FRC) were numerous in 4NQO-induced tumors (18) we investigated their abundance by staining for gp38 and ERTR7 (and the lack of LYVE1 staining). Indeed we observed FRC to be highly abundant in the OSCC13 tumors (**Figure 2D**). We noticed that ERTR7+/gp38+ cells surrounded the tumors and that the ERTR7 signal showed a continuation from the tumor border into the tumor mass reminiscent of FRC migrating from outside tissue into the tumor mass. FRC are an important source of matrix as demonstrated in the 4NQO-induced tumors (18). Here, we stained for TNC and saw a similar staining pattern as for ERTR7, separating tumor cells (p63+), supportive of FRC also expressing TNC in this model (**Figure 2E**). TNC was arranged in fibrillar matrix alignments resembling tumor matrix tracks (TMT) like in 4NQO-induced tongue and other tumors (12, 18, 38) (**Figure 2E**).

### Immune Suppressive TME in OSCC13 Tumors

**Figure 3:**
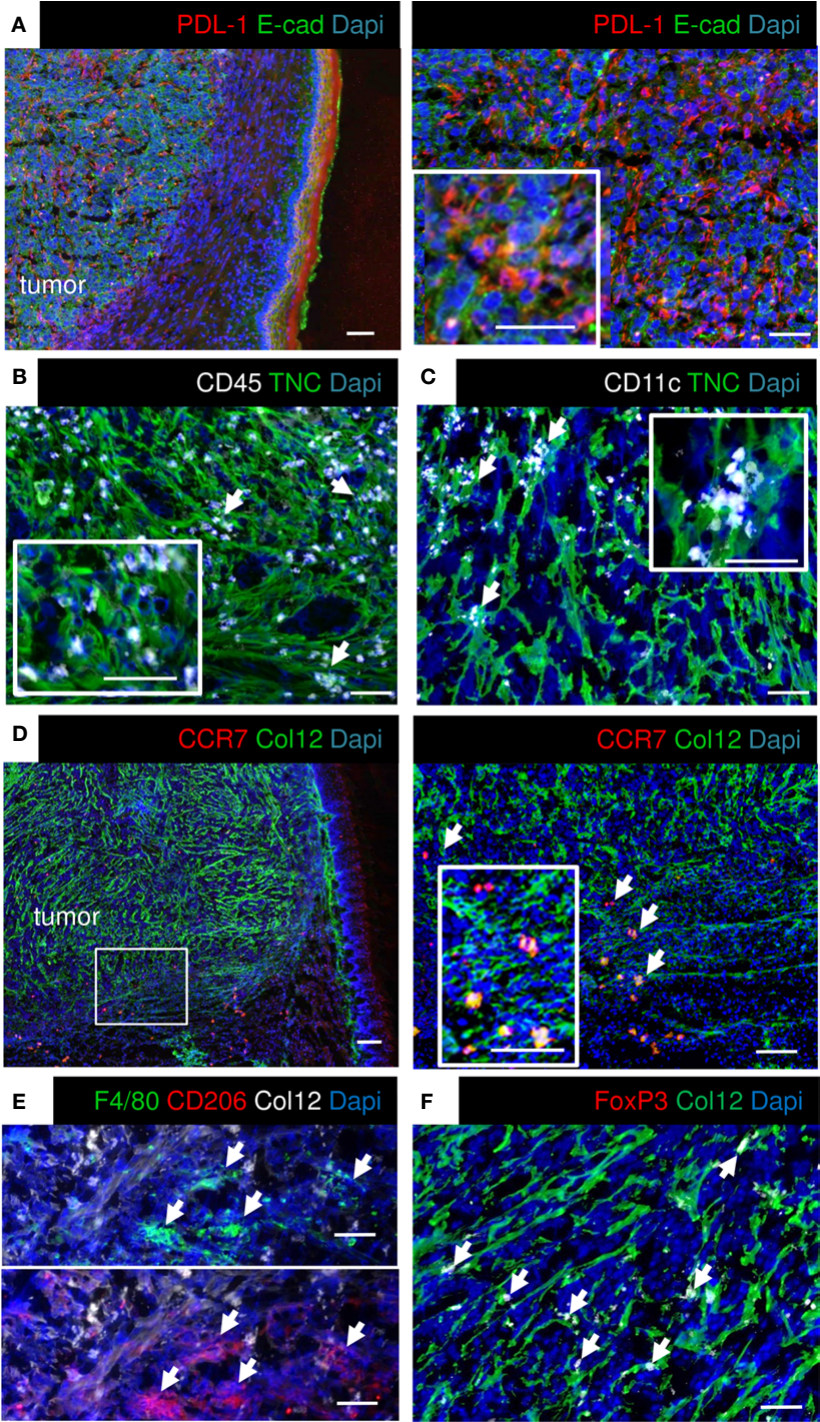
Characterization of immunity markers in OSCC13 tumors by immunostaining. About 4 weeks syngeneic tongue tumors derived from 1 × 10^6^ engrafted OSCC13 cells were stained for the indicated molecules. Representative images are shown (N = 3). Scale bar, 100 µm. Arrows point at CD45+ **(B)**, CD11c+ **(C)**, CCR7+ **(D)**, F4/80 and CD206+ **(E)** and Foxp3+ cells **(F)** that are in close vicinity to matrix. **(A, D)**, scale bar, 500 μm (left). Right panels, higher magnification. Scale bar, 50 μm **(A)**, 100 μm **(D)**.

As the RNA seq analysis showed that the cultured OSCC13 cells expressed *Cd274* we stained the OSCC13 tumors for PDL-1 and indeed found an ubiquitous expression, resembling high PDL-1 in human OSCC, 4NQO-induced OSCC and other OSCC grafted tumors [**Figure 3A** (24)]. Human OSCC and 4NQO-induced tumors are known to be highly invaded by tumor-infiltrating leucocytes (TIL). To address the immune status also in the OSCC13 tumors we stained for the leukocyte marker CD45+ and noticed an accumulation of leukocytes around and inside the tumors with close vicinity to TNC and laminin (LM) matrix tracks which is again similar to the 4NQO-induced OSCC [**Figure 3B** and **Supplementary Figure S1C** (18)]. Staining for CD11c+ myeloid/dendritic cells revealed localization inside the TNC-rich stroma, like in the 4NQO-induced OSCC [**Figure 3C** (18)]. By staining for Col12 (another prominent matrix molecule in OSCC) together with C–C chemokine receptor type 7 (CCR7) we observed a TMT-like organization of Col12 similar to the 4NQO tumors (18). Moreover, we noticed CCR7+ cells [dendritic cells, macrophages and naïve T cells (39)] invading the tumors (**Figure 3D**). Staining for CD206 and F4/80 revealed high colocalization of both markers inside the tumors, indicating abundance of macrophages with a M2 phenotype (**Figure 3E**). Finally, we assessed the potential presence of immune suppressive Treg by Foxp3 staining. Indeed, FoxP3+ cells were present in vicinity to Col12 (**Figure 3F**). In summary, engrafted OSCC13 cells gave rise to tumors with a TME comprising TNC, LM and Col12 matrix, abundant fibroblasts and immune cells that shared an immune suppressive phenotype with 4NQO-induced tumors and human HNSCC (18).

### OSCC13 Tumor Cells Spontaneously Disseminate and Home to the Local Lymph Nodes

**Figure 4:**
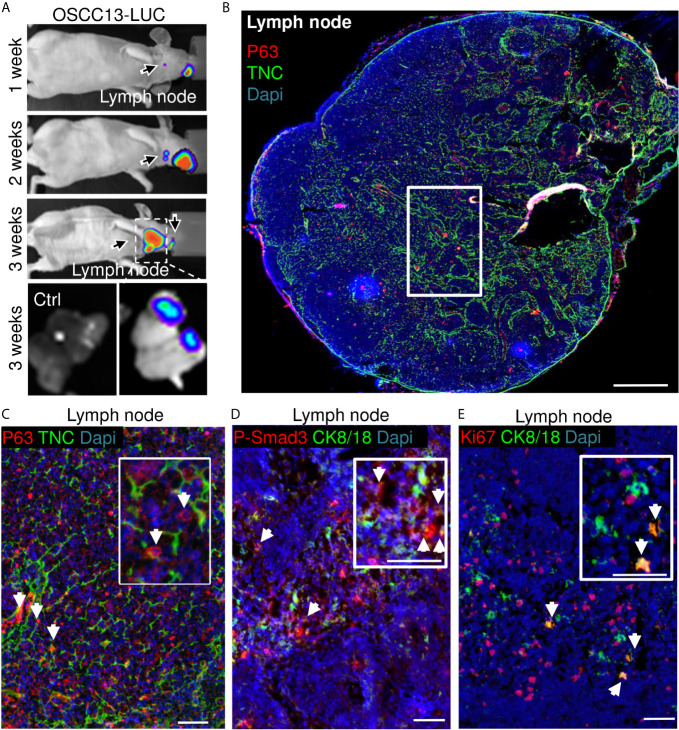
OSCC13 tumor cells home to the local lymph nodes and expand. **(A)** Detection of luciferase expressing OSCC13 cells in the lymph node by a Nightowl imaging machine in a nude mouse and in the dissected lymph nodes. Luciferin signal in the tumor and, expanding in the lymph node from 1–3 weeks. N = 3 mice. **(B–D)**, OSCC13 cells were engrafted in a C57Bl/6J mouse. **(B)** Representative result (N = 3) of disseminated OSCC13 cells in the lymph node by p63 **(C)** and P-Smad3 **(D)** staining and proliferating CK8/18+ tumor cells by Ki67 staining **(E).** Note tumor cells (p63+) to be placed in TNC+ matrix (inlet of **C**) and proliferation of tumor cells (CK8/18+) **(E)**, confirming tumor cell expansion in the lymph nodes presumably forming metastasis in the immune competent context like in nude mice **(A)**. N = 3 mice. Scale bar, 500 µm **(B)** and 50 µm **(C–E)**. Arrows point at tumor cells.

As tumor cells were found in the local lymph nodes of mice with 4NQO-induced tumors (18), we wondered whether OSCC13 cells were also able to spontaneously disseminate. First, we used a bioluminescence approach to detect OSCC13 cells engineered to express luciferase and monitored luciferin in tumors and local lymph nodes upon grafting of OSCC13 cells underneath the tongue mucosa of nude mice. We saw a bioluminescence signal in the primary tumor one week after grafting that increased with time (but was hidden at 3 weeks behind the anesthesia cap) (**Figure 4A**). More importantly, we also saw a small luciferin signal in the lymph nodes already one week after grafting that further increased by week 2 and was highly prominent at week 3. A strong luciferin signal was confirmed by imaging of the extracted lymph nodes demonstrating that the OSCC13 tumor cells had spontaneously homed to the lymph nodes and expanded there (**Figure 4A**). Next, we investigated potential lymph node metastasis in the immune competent condition by grafting OSCC13 cells into the tongue mucosa of a C57Bl/6J mouse followed by tissue staining of the lymph nodes 4 weeks after grafting. As we had seen P-Smad3 expression in OSCC13 tumors [**Figure 2B** (18)], we stained the lymph nodes for p63, in addition to other markers. Indeed in the lymph nodes of all investigated tumor mice (3/3) we saw numerous p63+ cells inside the lymph nodes (**Figure 4B**). Co-staining with TNC revealed that the p63+ tumor cells were present in association with TNC that was expressed in short fibrillar networks resembling TNC-rich reticular fibers in lymph nodes [**Figure 4C** (18)]. As tumor cells expressed P-Smad3 (**Figure 2B**) we used this marker together with CK8/18 and observed cells that were positive for both markers, further supporting the presence of OSCC13 tumor cells in the lymph nodes (**Figure 4D**). Next, we addressed whether tumor cells expanded in this model as seen in nude mice (**Figure 4A**) and used staining for the proliferation marker Ki67 in combination with CK8/18+. Indeed, we noticed CK8/18+ cells in the lymph nodes that also expressed Ki67 confirming that disseminated tumor cells could expand in the lymph nodes (**Figure 4E**). We wondered whether tumor cells potentially also homed to the lung in the grafting model, the second most frequent site of metastasis in the human OSCC patient (40). However, the experiment had to be discontinued after 4 weeks as the tumors had reached the ethically accepted size, thus a potential metastasis to the lung could not be addressed rigorously. Interestingly, in one tumor mouse we observed a macroscopical metastasis on the outer surface of the lung, suggesting that potentially tumor cells may also disseminate to the lung. Future studies have to address the lung metastatic potential of OSCC13 cells in more detail.

Altogether, these results strongly suggest that tumor cells from the OSCC13 engrafted tongue tumors spontaneously disseminated and homed to the local lymph nodes where some tumor cells proliferated demonstrating their metastatic potential.

### Response of OSCC13 Cells and Derived Tumors to Radiotherapy

**Figure 5:**
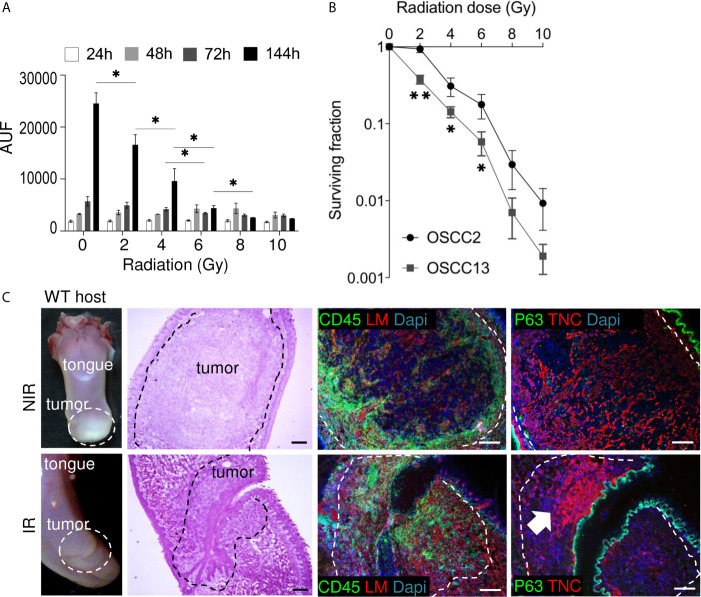
Impact of irradiation on cultured OSCC13 cells and tumors. **(A)** OSCC13 cell proliferation upon exposure to the indicated irradiation doses after the indicated time points (hours, h) using a resazurin assay. Data are represented as mean ± SEM of at least three independent experiments. Student–Newman–Keuls test. *p < 0.05. **(B)** Clonogenic survival assessment upon exposure to the indicated irradiation doses. The surviving fractions are represented. Mean ± SD of four independent experiments. ANOVA test. **p <0.001, *p < 0.005. **(C)** Representative (N = 5) macroscopical images of H&E stained tongue tissue from OSCC13 tumors in a WT host and upon immunostaining with specific antibodies against laminin (LM, red) or TNC (red) together with CD45 (green)or p63 (green). Scale bar, 100 µm. Arrow points at dense TNC matrix seen upon irradiation. IR, irradiated; NIR, non-irradiated.

Radiotherapy cannot only kill tumor cells but also triggers inflammation and changes in the tumor bed that may counteract tumor regression (41). So far immune competent murine grafting models with a relevant TME to address the roles of radiotherapy in OSCC were not described. To investigate radiosensitivity we exposed OSCC13 cells (in comparison to OSCC2 cells) to increasing single doses of irradiation and determined subsequent tumor cell proliferation and survival in clonogenic assays. We observed a significant reduction in proliferation of OSCC13 cells 6 days after irradiation already at 2 Gy (only 68% living cells) with a pronounced dose-dependent effect up to 10 Gy, respectively rendering only 10% cells alive after exposure to 10 Gy (**Figure 5A**). In comparison, a 2 Gy irradiation dose had no effect on proliferation of OSCC2 cells 6 days post-radiotherapy (**Supplementary Figure S2**). Cell survival of OSCC13 cells at 2 Gy was already significantly reduced compared to OSCC2 cells (surviving fraction at 2 Gy (SF2) was 0.38 and 0.94, respectively for OSCC13 and OSCC2) and decreased further, down to 0.2% and 0.9% for OSCC13 and OSCC2, respectively after 10 Gy (**Figure 5B** and **Supplementary Figure S2**). This experiment revealed that OSCC13 cells were more radiosensitive than OSCC2 cells. Therefore, we used OSCC13 cells in the following grafting and irradiation experiments.

Non-tumor bearing mice were irradiated with 2 Gy before collection of blood and enumeration of white and red blood cells 2 weeks later that indicated the absence of irradiation-induced toxicity (**Supplementary Figure S3A**). Therefore we used 2Gy to irradiate mice with OSCC13 cell (submucosal) engrafted tumors, collected the tongues 2 weeks later and investigated a potential impact of radiotherapy by tissue staining. Indeed, H&E staining revealed that a 2 Gy irradiation dose destroyed large tumor areas indicated by an altered tumor appearance and lower tumor cell density as seen by reduced H&E, p63 and DAPI staining (**Figure 5C**). We also noticed high CD45+ leukocyte abundance with different distribution patterns showing an accumulation at the tumor rim or, within the tumor center as clusters or as single cells (**Figure 5C**). Upon irradiation TIL appeared locally densely packed by a strong CD45+ signal (**Figure 5C** and **Supplementary Figure S3B**). As TNC can be induced by radiotherapy (42) we wondered whether 2 Gy irradiation had an impact on TNC expression. Whereas, TNC appeared as a regular and homogenous fibrillar network in non-irradiated (NIR) tumors as seen before (**Figure 2**), upon irradiation the TNC signal was largely gone (in destroyed tissue) or appeared locally as condensed matrix together with dense nuclei. We propose that thick TNC matrix and cell dense areas represent tissue that has not been killed by irradiation (**Figure 5C** and **Supplementary Figures S3B, C**).

### Impact of TNC on Immunity and Radiotherapy-Induced Tumor Regression

**Figure 6:**
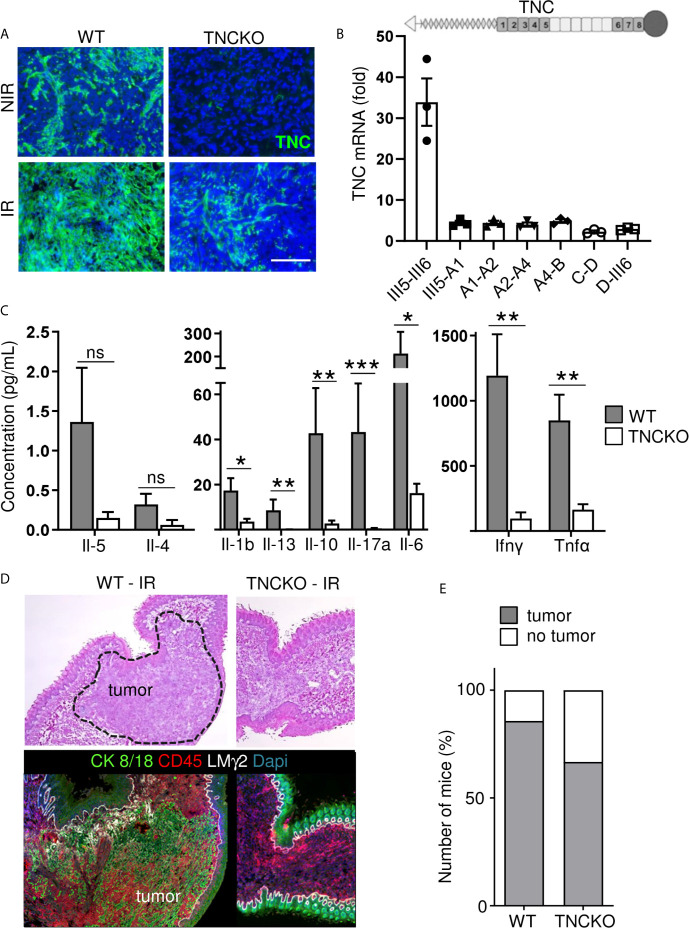
Impact of TNC on T cell stimulation and on OSCC13 tumor abundance upon irradiation. **(A)** TNC immunostaining of non-irradiated (NIR) and irradiated (IR) tumors from WT and TNCKO mice 2 weeks upon irradiation with 2 Gy. Scale bar 100 µm. WT, N = 6, TNCKO, N = 9. **(B)** Gene expression analysis (qRTPCR) of different TNC FNIII domains (exon junctions) in OSCC13 cells 24 h upon 2 Gy irradiation (normalized to GAPDH). N = three experiments. The domain organization of TNC is schematically depicted on top with heptad oligomerization domain (triangle), FNIII domains (dark, constant, light, alternatively spliced) and fibrinogen globe (circle). **(C)** Expression of cytokines in T cells present in OSCC13 tumors from WT and TNCKO mice (2 weeks upon engraftment), 24 h after stimulation with CD3/CD28 beads, with a cytokine cytometry bead array (CBA) assay. Mean ± SEM, Mann–Whitney test, *p < 0.05, **p < 0.01, ***p < 0.05. **(D)** Representative H&E and immunostaining images for CK8/18 and CD45 (N = 5) of a non-irradiated and irradiated tongue from a OSCC13 tumor bearing mouse (WT or TNCKO). **(E)** Detection of tumors (by CK8/18 signal) in irradiated WT (N = 6/7) and TNCKO mice (N = 6/9); ns, not significant. Note that whereas all engrafted mice developed tumors in both genotypes, upon irradiation there was a partial regress.

In a tumor TNC is mostly expressed by stromal cells, but can also be expressed by immune and tumor cells (43). We used OSCC13 tumor cell grafting into a WT or TNCKO host, respectively to address the origin of TNC. We observed no TNC in tumors of a TNCKO host indicating that tumor cells poorly express TNC. Upon irradiation with 2 Gy, TNC levels increased in the irradiated tumors both in the TNCKO and WT context, however much less in the TNCKO host suggesting that the stromal cells were the major source of TNC (**Figure 6A**). Apparently, 2 Gy radiotherapy also triggered some TNC expression in the tumor cells as seen by a TNC signal in the TNCKO tumors (**Figure 6A**). As TNC can be expressed in different isoforms, we used exon junction specific primers to determine by qRTPCR whether radiotherapy had an impact on the TNC isoforms expressed by the tumor cells. Indeed cultured OSCC13 cells expressed some higher molecular weight TNC isoforms 24 h after irradiation as all primers amplified the respective alternatively spliced fibronectin type III (FNIII) domains, however the short form of TNC without the extra FNIII domains was the prominent form (**Figure 6B**). As in tumors the large isoform of TNC is highly expressed and was shown to be associated with an inflammatory pro-tumorigenic function (44) future studies have to address the potential roles of the different forms of TNC in the irradiated tumor tissue.

Next we investigated whether TNC had an impact on immunity. Therefore we investigated the abundance of different immune subtypes by flow cytometry. We observed that CD4+ and CD8+ TIL were more abundant than NK and B cells. However, no difference between tumor genotypes was seen (**Supplementary Figure S4A**). Next, we lysed the tumors and triggered T cells with a mixture of CD3 and CD28 antibodies and determined expression of cytokines 24 and 72 h later, using a cytokine cytometry bead array (CBA) assay, respectively. This analysis revealed an induction at both time points and a clear difference between tumor genotypes. Stimulated T cells from TNCKO tumors expressed less Interleukin 1 beta (IL1β), IL10, IL17A, IL6, IFNγ and tumor necrosis factors α (TNFα) whereas no difference for IL4 nor IL5 was seen that were poorly expressed (**Figure 6C** and **Supplementary Figure S4B**). These results indicated that the TME in tumors of a TNCKO host may have a less inflammatory TME with less active T cells.

To address whether TNC impacted irradiation-induced tumor regression, we applied 2 Gy to OSCC13 tumors grown in a WT or TNCKO host. By staining for Dapi and CK8/18 we investigated the irradiated tissue and noticed that in the TNCKO host 30% of mice showed complete tumor regression which was less prominent in the WT host (14%) (**Figures 6D, E**). In all other tumors we determined the tumor areas by H&E staining before and after irradiation. We noticed that tumors of both genotypes partially regressed upon irradiation but that the host genotype did not have an impact on the tumor size (measured as surface) in the given short time frame of the experiment (**Supplementary Figure S4C**).

### Impact of Irradiation and TNC on CD45+ and CD206+ Immune Cell Infiltration

We compared the localization and abundance of leukocytes in OSCC13 tumors upon growth in a TNCKO host with that in a WT host by IF imaging. This showed abundant CD45+ leukocytes also in the TNCKO context within the tumors and in their direct vicinity (**Supplementary Figures S4B, D**). Upon irradiation CD45+ leukocytes also infiltrated the TNCKO tumors. In contrast to this broad infiltration in the TNCKO tumors this appeared to be more local in the WT tumors (**Supplementary Figure S4D**).

**Figure 7:**
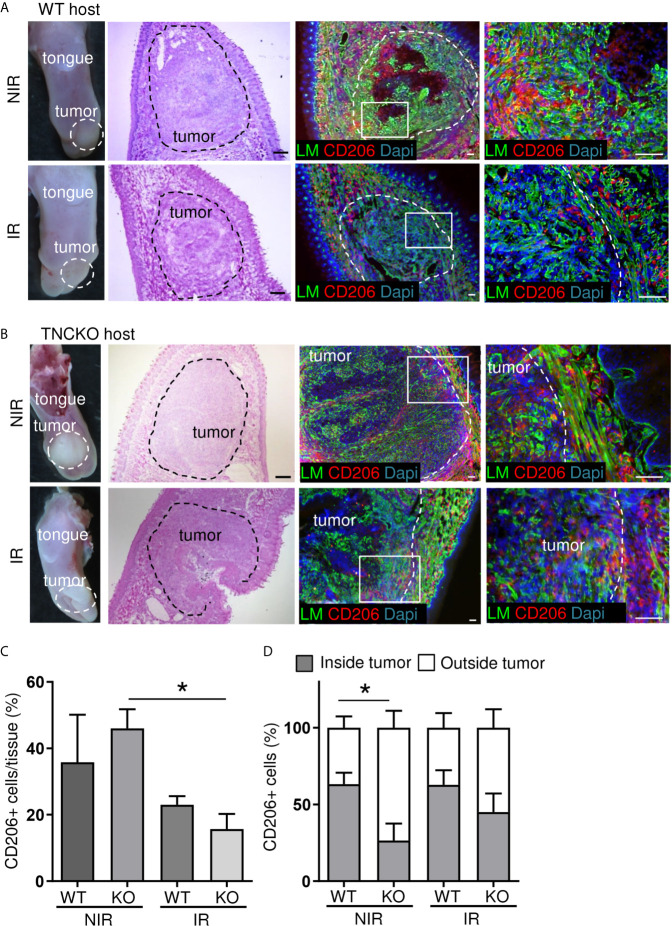
Irradiation effects on CD206+ macrophages in OSCC13 tumors. Representative (N = 5) macroscopical images of H&E stained tongue tissue and immunostaining with specific antibodies against laminin (LM, green) and CD206 (red) in OSCC13 tumors from WT **(A)** and TNCKO mice **(B)**. Scale bar, 100 µm. **(C)** Quantification of CD206+ cells in the tongue (referenced to DAPI signal) in percentage. N = 3, mean ± SEM, non-parametric ANOVA followed by Dunns post-test, *p < 0.05. **(D)** Relative distribution of CD206+ cells inside or outside the tumor of WT and TNCKO mice in non-irradiated (NIR) and irradiated (IR) conditions, respectively. N = 3, mean ± SEM, non-parametric ANOVA followed by Dunns post-test, *p <0.05. NIR, non-irradiated mice; IR, irradiated mice.

As we previously described an impact of TNC on CD206+ pro-tumoral M2 macrophages in the syngeneic NT193 breast cancer grafting model (45) we investigated the abundance and spatial distribution of CD206+ by tissue staining and subsequent quantification (**Figures 7A–D**). This experiment revealed numerous CD206+ macrophages inside the tumors and in direct tumor vicinity (**Figures 7A, B**). Whereas no difference in CD206+ macrophages was seen in WT tumors with and without irradiation TNCKO tumors showed a tendency towards less CD206+ cells after irradiation (**Figure 7C**). Determining the spatial distribution of CD206+ cells we noticed a higher inside-to-outside ratio of CD206+ macrophages in WT than TNCKO tumors. Irradiation did not impact this ratio in the given time frame (**Figure 7D**).

Altogether we have established a novel syngeneic OSCC grafting model derived from a 4NQO-induced tongue tumor that showed similarities with human OSCC by recapitulating mutations seen in human tumors, constitutive TGFβ signaling, abundant TIL within TNC-rich stroma and, spontaneous tumor cell dissemination to and expansion in the local lymph nodes. Most importantly, this model was sensitive to a 2 Gy dose of irradiation and allows not only to investigate tumor cell killing by radiotherapy but may be also useful to investigate potential rebound effects where induction of TNC could function as marker for adverse responses.

## Discussion

Radiotherapy is the most frequent treatment of patients with OSCC apart from surgery. Although radiotherapy kills the tumor cells, tumors relapse frequently and patients manifest with secondary tumors causing high morbidity and frequent metastasis to the local lymph nodes and lung, altogether resulting in a low 5-year survival (1). Radiotherapy and immune checkpoint therapy applied in human HNSCC cancer patients are important however, they often have either low efficiency or strong side effects (46). Therefore, novel immune competent tumor models recapitulating HNSCC-specific mutations and HNSCC-specific TME parameters are needed to better understand the targeting actions, to improve established targeting regimens and to develop novel targeting approaches.

Here we have developed a novel immune competent tongue OSCC tumor grafting model that we have investigated in detail by mutation and gene expression analysis, flow cytometry, cytokine expression analysis, extensive tissue staining and loss of function analysis. This comprehensive information on the TME and mutation phenotype has not been provided on previously published models. Mutation analysis revealed that our OSCC13 cells present mutational patterns and nucleotide substitution exchanges that recapitulate both the characteristics of tobacco- and 4NQO-induced tumor signatures. Moreover, OSCC13 cells present mutations on genes also frequently mutated in human tumors and in particular mutations in the Trp53 gene common to human HNSCC. Transcriptomic analysis of OSCC13 cells also revealed similarities in activation of signaling pathways (including TGFβ) like in many human HNSCC. Altogether the mutation pattern and gene expression profile of the OSCC13 cells is more similar to human HNSCC than other published models (24).

Although we did not detect tumor vascularization presumably due to the short duration of the experiment, important events seen in 4NQO-induced tumors were recapitulated in this model such as the development of a structured stroma with abundant matrix and TIL infiltration. We noticed effects on tumor immunity that can be considered to be anti-tumorigenic (e.g. abundant infiltration of macrophages, CD11c+ myeloid/dendritic cells, CD4+ and CD8+ TIL cells) but also pro-tumorigenic (e.g. high abundance of FRC as potential source for deposited matrix, co-localization of immune subtypes with matrix (reminiscent of retention by the matrix), numerous immune suppressive CD206+ M2 macrophages and Foxp3+ T reg cells and high PDL-1). Thus, this model could be suitable to investigate immune evolution in context of radiotherapy and/or drug targeting. Moreover, the OSCC13 tumor cells spontaneously disseminated to the local lymph nodes where they expanded demonstrating a high metastatic capacity. Whereas metastatic properties of murine OSCC cells were previously documented in a nude mouse model (47) here we described tumor cell dissemination and proliferation in the lymph nodes of immune competent OSCC13 tumor mice.

As the tumor cells expressed high PDL-1 and CTLA4 the syngeneic OSCC13 grafting model may be amenable for targeting immune checkpoints in particular in combination with radiotherapy as we have shown that OSCC13 cells and tumors are radiosensitive. Whereas irradiation experiments with human OSCC cells have previously been published in immunodeficient mice (48) to our knowledge OSCC grafting models in an immune competent host have not yet been developed for radiotherapy research. In our novel model, we have shown that OSCC13 tumors partially regressed upon exposure to 2 Gy. This model is also useful to address the effects of radiotherapy on tumor immunity as tumor cells are grafted in the tongues of immune competent mice. We propose that this model may be useful for a future investigation of rebound effects (potentially enhancing lymph node metastasis) as we have seen that 2 Gy upregulated TNC. As we saw an impact of radiotherapy on dense TNC matrix expression and TIL infiltration, we speculate that TNC could serve as indicator for an immune-suppressive TME enforced by irradiation, potentially promoting tumor regrowth in the future. Here, several pathways could be involved as we saw that TNC promoted conversion of macrophages into a pro-tumoral M2 phenotype involving Toll Like Receptor 4 (TLR4) and activated C-X-C chemokine receptor type 4 (CXCR4) signaling causing immobilization of CD8+ T cells in the stroma thereby inhibiting anti-tumor immunity (17, 45, 49). Moreover, TNC profoundly shaped the TME enhancing Chemokine (C–C motif) ligand 21 (CCL21)/CCR7 signaling thereby immobilizing CD11c+ myeloid cells thus impairing their function. As inhibition of TLR4, CXCR4 and CCR7 impacted anti-tumor immunity and reduced tumorigenesis (tumor numbers, growth and metastasis) (17, 18, 45), our novel OSCC13 grafting model may be suitable to investigate these and other alternative targeting approaches in particular in combination with radiotherapy.

Altogether, we have generated a novel immune competent OSCC grafting model recapitulating important properties of human HNSCC including HNSCC-relevant mutations, an immune-suppressive TME and radiosensitivity which opens novel opportunities for future targeting the TME and in particular the matrix in conjunction with radiotherapy and perhaps immune checkpoint targeting drugs.

## Data Availability Statement

The original contributions presented in the study are included in the article/**Supplementary Material**. Further inquiries can be directed to the corresponding authors.

## Ethics Statement

The animal study was reviewed and approved by CREMEAS.

## Author Contributions

CS, TL, HB, CL, LP, AY, GR, CJ, CA-F, CA, FS, and NP performed experiments and analyzed data. CS, TL, HB, and GO wrote the manuscript. GN, FA, and RC supervised experiments. CS and GO designed the study, analyzed data, and revised the manuscript. NS assisted the manuscript revision. All authors contributed to the article and approved the submitted version.

## Funding

This work was supported by INCa (FITMANET, PAIR-VADS11-023) and INCa/LNCC ECMpact (AAP2017.LNCC), ANR (AngioFib), Ligue contre le cancer (CCIRGE), University Strasbourg and INSERM to GO, Aviesan ITMO cancer (Radio-3R) to GO and GN, AAP2017.LNCC (FA), MRT fellowship to AY and fellowship from the Chinese Scholarship Council to CL.

## Conflict of Interest

The authors declare that the research was conducted in the absence of any commercial or financial relationships that could be construed as a potential conflict of interest.

## Abbreviations

4NQO, 4-nitroquinoline-1-oxide; AUF, arbitrary units of fluorescence; CBA, cytometry bead array; CCL21, Chemokine (C–C motif) ligand 21; CCR7, C–C chemokine receptor type 7; CXCR4, C-X-C chemokine receptor type 4; DC, dendritic cells; ECM, the extracellular matrix; EMT, epithelial-to-mesenchymal transition; FNIII, fibronectin type III; FRC, fibroblast reticular cells; H&E, hematoxylin and eosin; HNSCC, head and neck squamous cell carcinoma; HPV, human papillomavirus; IF, immunofluorescence; IFNγ, Interferon γ; IL1β, Interleukin 1β; IR, irradiated; LM, Laminin; NIR, non-irradiated; ORA, over-representation analysis; OSCC, oral squamous cell carcinomas; PE, plating efficiency; PFA, paraformaldehyde; SF, surviving fraction; TGFβ, transforming growth factor beta; TIL, tumor infiltrating leukocytes; TLR4, Toll Like Receptor 4; TME, tumor microenvironment; TMT, tumor matrix tracks; TNC, tenascin-C; TNFα, tumor necrosis factors α.
