# Supplementary material for: Impact of Tenascin-C on Radiotherapy in a Novel Syngeneic Oral Squamous Cell Carcinoma Model With Spontaneous Dissemination to the Lymph Nodes
Source: Front Immunol. 2021 Jul 5;12:636108. doi: 10.3389/fimmu.2021.636108 (PMC8287883; doi:10.3389/fimmu.2021.636108)
Supplement: Supplementary file 1 [file DataSheet_1.pdf]

Supplementary information to “Impact of tenascin-C on radiotherapy in a novel syngeneic oral squamous cell carcinoma model with spontaneous dissemination to the lymph nodes by Spenlé and collaborators

Supplementary Figures S1 – S4

Supplementary Tables S1 – S3

Figure S1

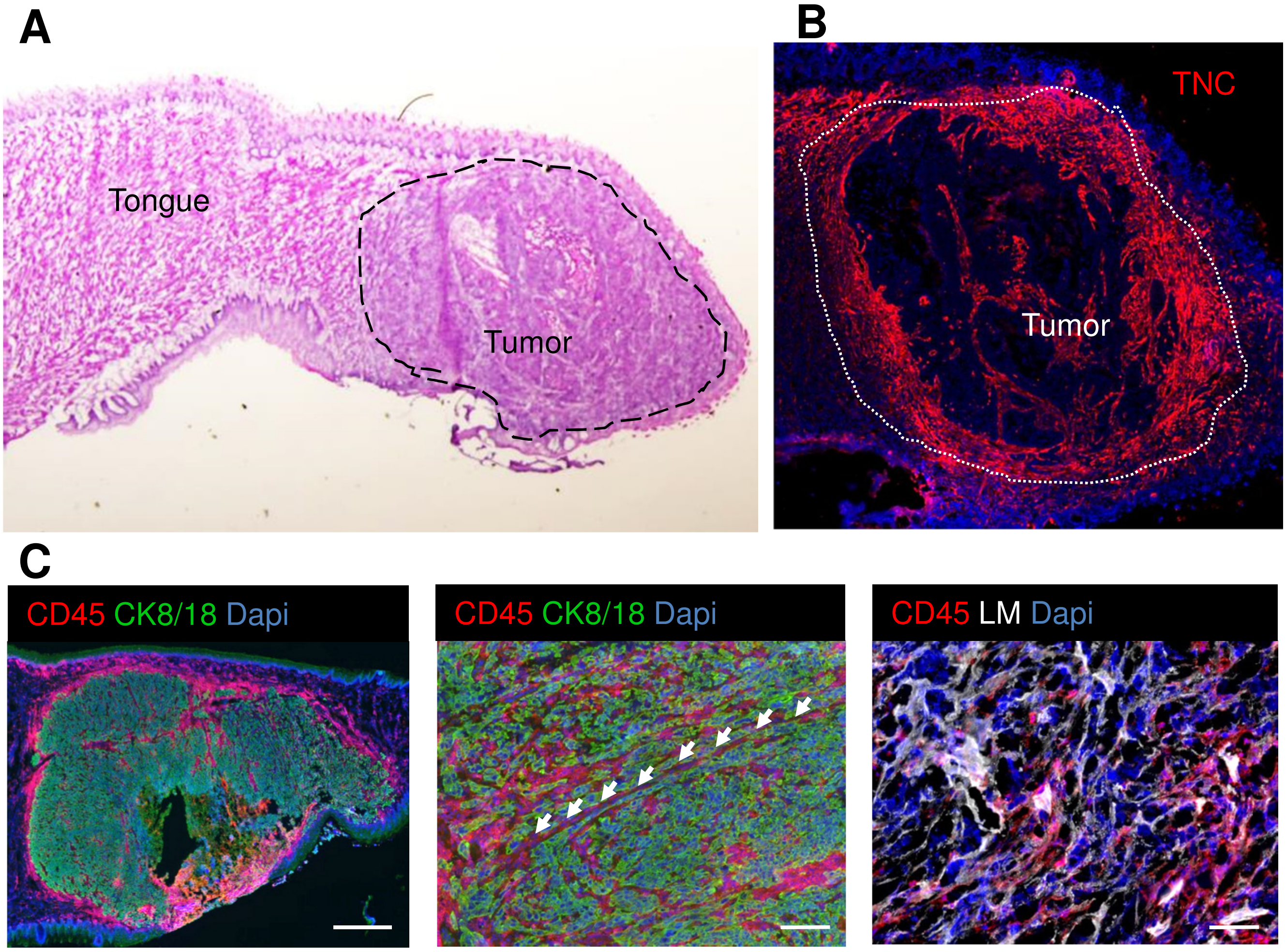

Figure S1 Overview of OSCC13 tumor morphology and candidate expression Hematoxylin-eosin staining of a sagittal slide of a tongue with a grafted OSCC13 tumor **(A)** and TNC immunostaining **(B)**. **(C)** OSCC13 tumor tissue staining for the indicated proteins. White arrows points at CD45 positive cells in area where Ck8/18 is absent and field with matrix tracks. Scale bar, 500µm (left) or 100 µm (middle and right).

Figure S2

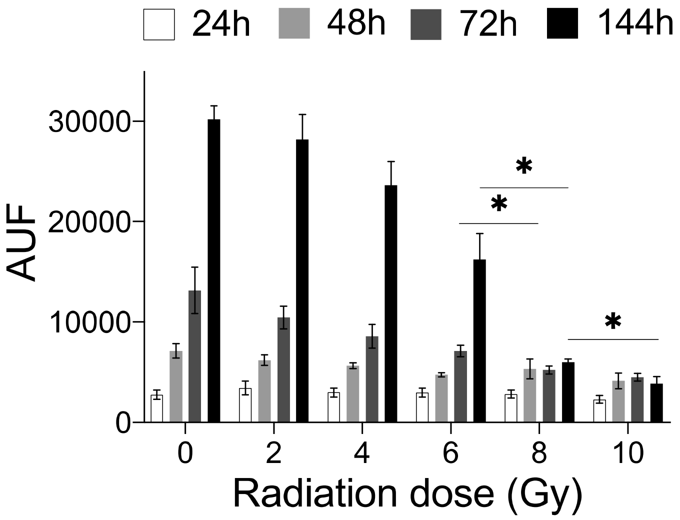

Figure S2 Impact of 2 Gy irradiation on cultured OSCC13 tumor cells Cell proliferation assay of OSCC2 cells treated with increasing dose of irradiation. N = 4, Student-Newman-Keuls test *p < 0,05.

Figure S3

A

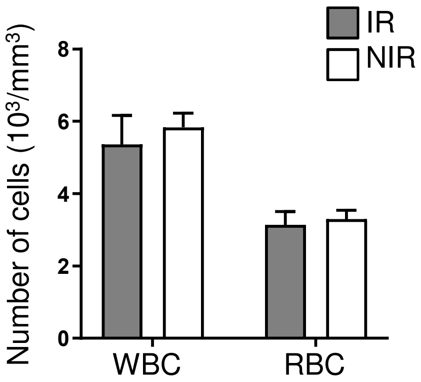

Figure S3 Impact of irradiation on toxicity in non-tumor bearing mice and on p63 and TNC expression in OSCC13 tumors

(A) Enumeration of white blood cells (WBC) and red blood cells (RBC) in non-irradiated (NIR) or irradiated (IR) mice, N = 5. Mann-Whitney test. **(B)** Images of NIR and IR OSCC13 grafted tongue tissue in macroscopical view, upon hematoxylin-eosin staining and immunostaining for TNC (red) and p63 (green) as indicated. Bold arrows point at strong TNC expression and thin arrows indicate p63 positive cells inside the tumor. Numbers indicate the sample identity. Scale bar, 200 µm. **(C)** TNC immunostaining in non-irradiated (NIR) and irradiated (IR) OSCC13 tumors. Areas of dense TNC expression are compared between tumors. Numbers indicate the sample identity. Scale bar, 20 µm.

Figure S3

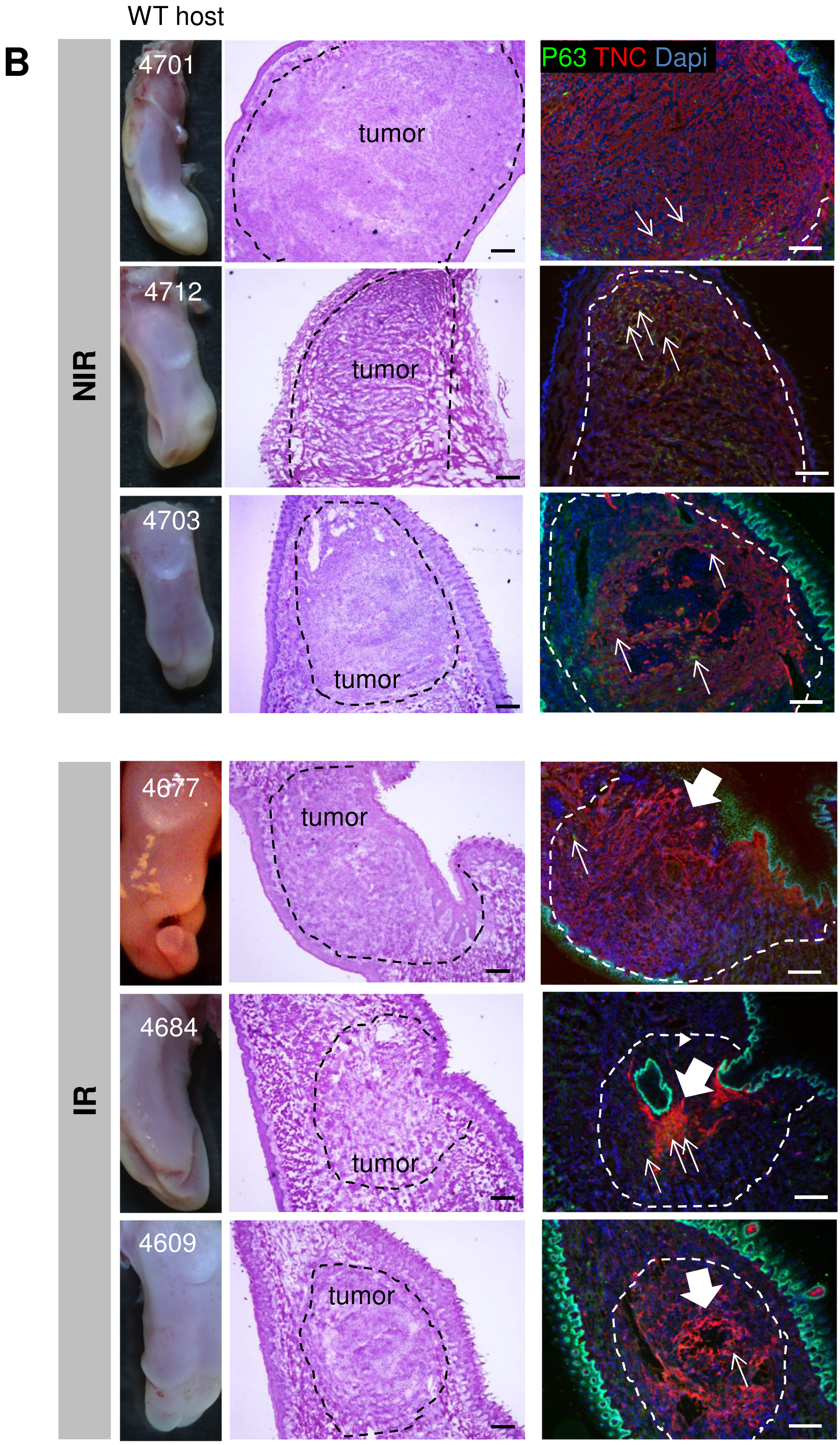
WT host
B
NIR
IR
4701
4712
4703
4677
4684
4609
tumor
P63 TNC Dapi

Figure S3

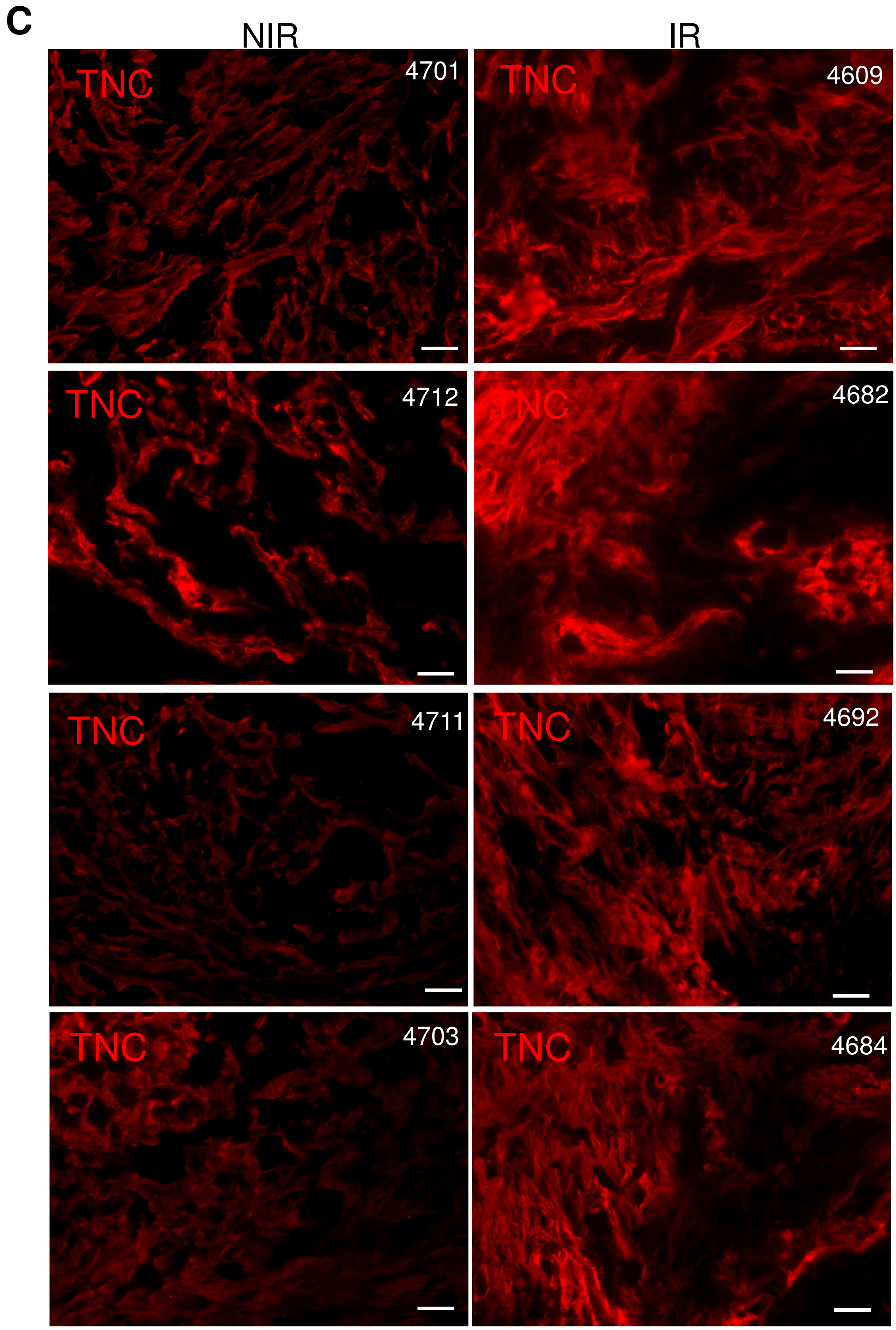
C
NIR
IR
TNC
4701
TNC
4609
TNC
4712
TNC
4682
TNC
4711
TNC
4692
TNC
4703
TNC
4684

Supplementary Figure S4

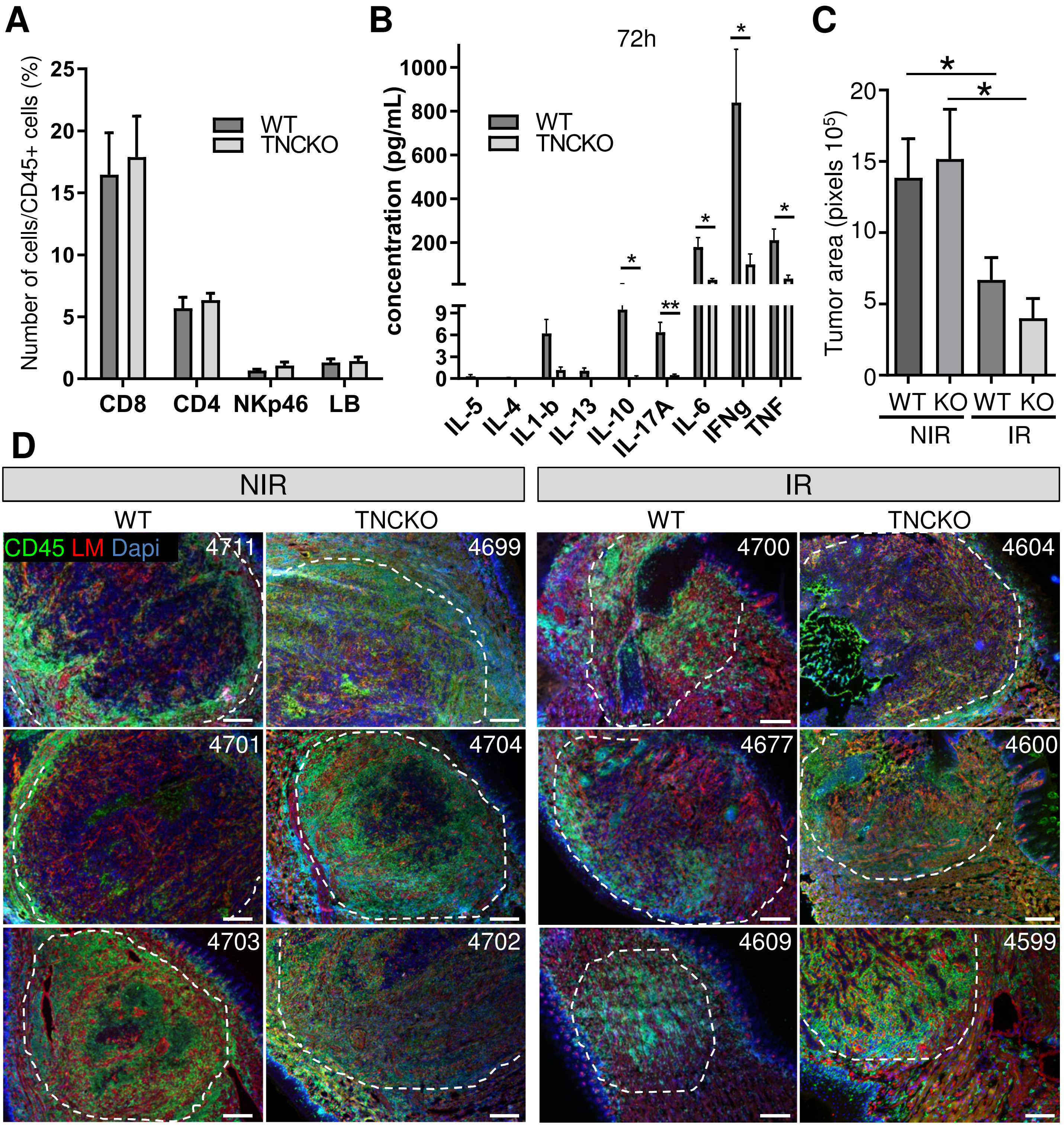

Figure S4 Impact of irradiation on tumor size and immune cell infiltration
(A) Number of lymphoid subsets per CD45+ Leukocytes in OSCC13 tumors of WT and TNCKO mice determined 2 weeks after engraftment. N=7 Mean ± SEM, Mann-Whitney test. **(B)** Measurement of cytokines 72 hours after stimulation. WT, N = 7; TNCKO, N = 5. Mean ± SEM, multitude unpaired t-test, * $p < 0.05$. ** p<0,005. **(C)** Tumor area of OSCC13 tumors in WT and TNCKO mice in non-irradiated (NIR) or irradiated (IR) conditions. NIR, N = 4; IR, N = 7. Mean ± SEM, non-parametric ANOVA followed by Dunns post-test, * $p < 0.05$. **(D)** Images of NIR and IR OSCC13 grafted tongue tissue upon immunostaining for pan-Laminin (LM, red) and CD45 (green). Numbers indicate the sample identity. Scale bar, 200 µm. Note that samples 4700 and 4711 were shown in Fig. 6C and are here displayed for comparison.

Table S1 Antibody list for cell and tissue staining

Name	Host	Reference	Source
E-cadherin	rat	ECCD-2	Novex
Vimentin	rabbit	EPR3776	Epitomics
Tenascin-C	rat	MTn12	G. Orend
Pan LM	rabbit	Ln6 7s	Simo et al., 1992
ERTR7	rat	Clone 51824	Abcam
p63	rabbit	NP_003713	Abcam
CD45	rat	Clone 30-F11	BD Pharmingen™
CD31	rat	Clone MEC 13.3	BD Pharmingen™
Ck8/18	Guinea pig	Clone GP11	Progen
Gp38	Guinea pig	Clone 8.1.1	Thermofisher
CD11c	Armenian Hamster	Clone HL3	BD Pharmingen™
Foxp3	rat	Clone FJK-16s	Thermofisher
PDL-1	rat	EPR19759	Abcam
Ki67	rabbit	Clone Sp6	Thermofisher
Lyve-1	rabbit	Clone 103-PA50	ReliaTech GmbH
CCR7	rat	Clone 4B12	R&D system
Col12	rabbit	Clone Kr33	DR. M Koch
CD206	rat	C068C2	Biolegend
F4/80	rat	CI:A3-1	Abcam
Anti-rat Alexa488	goat	Alexa Fluor® 488 AffiniPure Goat Anti-Rat IgG (H+L)	Jackson Immunoresearch
Anti-rabbit Cy3	goat	Cy™3 AffiniPure Goat Anti-Rabbit IgG (H+L)	Jackson Immunoresearch
Anti-mouse Alexa488	goat	Alexa Fluor® 488 AffiniPure Goat Anti-Mouse IgG (H+L)	Jackson Immunoresearch
Anti-Armenian Hamster Cy3	goat	127-005-160	Jackson Immunoresearch
Anti-guinea Pig Cy5	goat	706-165-148	Jackson Immunoresearch
Anti-rat IgG	goat	A11006	Jackson Immunoresearch

Table S2 Primer list

Gene	Primers	Sequence
TNC alternative exon junctions	*III5 – A1*	5'CAGGGCAAGAATACACTGTTCTCC3'<br>5' CCAGTTGAGTCTGAGGCCAT3'
TNC alternative exon junctions	A1 – A2	5' CCAGGCCTCAAGGTTGCC3'<br>5' GTCGTGTCAGCCTCTAGCAC3'
TNC alternative exon junctions	A2 – A4	5' GCCTGGGCTCAAAGCAGC3'<br>5' TCCAGTTGAGTGTGAGGCCG3'
TNC alternative exon junctions	A4 - B	5' GGCCTCAAGGCCGACAC3'<br>5' CATGTCGAAGATCCCGTCGG3'
TNC alternative exon junctions	C-D	5' GGCCTCATAACTGGCATTGG3'<br>5' TCAGCAGTCCAGGACAGACG3'
TNC alternative exon junction	D-III6	5' CCAGGGACATAACAGGTCTCAG3'<br>5' GGAAACTCTCCACCTGAGCAG3'
TNC alternative exon junctions	III5-III6	5' CAGGGCAAGAATACACTGTTCTCC3'<br>5' GGAAACTCTCCACCTGAGCAG3'
GAPDH	TaqMan probe	Roche

Table S3 Gene expression in OSCC13 cells (I)

Wnt signaling pathway (GO:0016055)

GENE NAME
Pten
Fzd9
Znrf3
Rnf138
Daam1
Fzd5
Wnt7b
Tnks
Bcl9l
Pygo2
Csnk1a1
Rnf146
Scyl2
Celsr2
Chd8
Fzd1
Zbtb33
Spin1
Krt6a
Lgr6
Cd44
Tmem198
Ryk
Axin1
Mark2
Lmbr1l
Tcf3
Dixdc1
Cpe
Csnk2a2
Csnk1e
Btrc
Csnk2a1
Nlk
Mesd
Lgr4
Ddb1
Zranb1
Amotl2
Pitx2
Myc
Cav1
AC139513,Celsr1
Macf1
Dvl2
Tle3
Prkaa1
Vangl2
Senp2
Ddx3x,Erh
Amotl1
Csnk1d
Usp34
Grk6
Sdc1
Ccnd1
Tax1bp3
Pias4
Wnt7a
Csnk1g1
Fzd6
Ccdc88c
Fbxw11
Fermt2
Tnks2
AC164883
Lzts2
Rab5a

Regulation of Wnt signaling pathway (GO:0030111)

GENE NAME
Sulf2
Fzd9
Fgfr2
Znrf3
Arntl
Rnf213
Ppm1a
Tnks
Wnk1
App
Jade1
Ctnnd1
Mvp
Ubr5
Csnk1a1
Rnf146
G3bp1
Scyl2
Pin1
Nrarp
Dapk3
Ppm1b
Lrrk1
Chd8
Fzd1
Mapk14
Spin1
Ift80
Lgr6
Gli3
Src
Tmem198
Limd1
Rapgef1
Axin1
Mllt3
Lmbr1l
Snx3
Dixdc1
Foxo1
Gsdma3
Csnk1e
Btrc
Csnk2a1
Nlk
Jrk
Hmga2
Lgr4
Zranb1
Abl2
Cdh1
Smad3
Sox9
Gnaq
Cav1
Lats1
Usp47
Smarca4
Macf1
Sox4
Tle3
Fermt1
Atp6ap2
Vangl2
Ddx3x,Erh
Csnk1d
Usp34
Lats2
Taz
Hdac1
Sdhaf2
Vcp
Itga3
Abl1
Nfkb1
Tax1bp3
Stk4
Csnk1g1
Fzd6
Yap1
Ccdc88c
Sema5a
Tnks2
AC164883
Kpna1
Lzts2
Vps35
Ilk

Table S3 Gene expression in OSCC13 cells (II)

Response toTGFβ signaling pathway (GO:0071559)

**GENE NAME**	Nrros	Nlk	Runx1
Smad2	Usp15	Dbn1	Col4a2
Ppm1a	Trp53	Pdcd5	Creb1
Fyn	Twsg1	Smad3	Smad7
Ovol2	Zfp36l1	Sox9	Gcnt2
Crk	Igf1r	Ptprk	Tgfbr3l
Dnmt1	Src	Cav1	Fermt2
Fut8	Map3k7	Hipk2	Ltbp3
Zfp36l2	Skil	Stk16	Eng
Scx	Zfhx3	Ptk2	Rock2
Nr3c1	Appl1	Hyal2	Zmiz1
Pxn	Jun	Ltbp1	Mtmr4

Regulation ofTGFβ signaling pathway (GO:0017015)

**GENE NAME**	Strap	Smad3	Hsp90ab1
Cav2	Skil	Cav1	Hspa5
Gipc1	Lemd3	Hipk2	Itga3
Smad2	Axin1	Fermt1	Rnf111
Ldlrad4	Xbp1	Glg1	Smad7
Snx25	Smurf1	Snw1	Tgfbr3l
Ppm1a	Furin	Ep300	Sp1
Bcl9l	Atl2	Zbtb7a	Eng
Npnt	Snx6	Adam17	Pmepa1
Pin1	Pdpk1	Fam89b	
Nrros	Thbs1	Zfp451	
Trp53	Smurf2	Ltbp1	

Supplementary Table S3 Gene expression in OSCC13 cells
Partial list of genes expressed in cultured OSCC13 cells as determined by RNA seq analysis. Here, genes involved in Wnt and TGFβ signaling are listed. All genes are deposited under accession number x (acquisition in progress).
